# Convergent and Construct Validity of a Conversation Difficulties Outcome Measure in Primary Progressive Aphasia

**DOI:** 10.1093/geroni/igab046.3679

**Published:** 2021-12-17

**Authors:** Haylie Santos, Nathan Gill, Elizabeth Salley, Hui Zhang, Emily Rogalski, Angela Roberts

**Affiliations:** 1 Northwestern University, Evanston, Illinois, United States; 2 Northwestern University, Chicago, Illinois, United States

## Abstract

Cognition and language changes, and their impacts on functional communication, are central to many dementias. Thus, functional communication, including conversation difficulties, is an important endpoint for clinical trials. To develop robust outcomes in primary progressive aphasia (PPA), a dementia characterized by communication impairments, we examined the convergent and construct validity of the Perception of Conversation Difficulties-Dementia Alzheimer’s Type (PCI-DAT; Orange et al., 2009). The PCI-DAT is a care partner reported measure of conversation difficulties. Eighty-two care partners with a mean age of 64.8 years (SD=10.61; 85% spouses, 5% adult children, 10% friends/siblings) whose mean relationship duration to the person with PPA was 39.1 (SD=15.1) years completed the study. Pearson’s correlation indicated a significant, modest correlation (r=-0.54, p<0.0001) between the PCI-DAT Perception of Conversation Difficulties subscale and the Communication Effectiveness Index (Lomas et al., 1989) suggesting strong convergent validity. A Rasch analysis conducted on the same PCI-DAT subscale showed high person (0.92) and item (0.95) reliability indicating a robust overall scale structure that adequately evaluates various levels of conversation difficulty severity in PPA. Six items (27%) had minor ‘fit’ issues (defined by Wright and Linacre, 1994 as having infit statistics < 0.6 or > 1.4) relative to the underlying construct. Results suggest strong convergent and construct validity of the PCI-DAT in PPA and indicate items that will benefit from further development. Overall, our results suggest that the PCI-DAT holds promise for use as a functional communication endpoint in PPA clinical trials. Data for all five PCI-DAT subscales will be presented.

